# Heart rate variability responses to cognitive stress in fibromyalgia are characterised by inadequate autonomous system stress responses: a clinical trial

**DOI:** 10.1038/s41598-023-27581-9

**Published:** 2023-01-13

**Authors:** Teemu Zetterman, Ritva Markkula, Teemu Miettinen, Eija Kalso

**Affiliations:** 1 Department of Anaesthesiology, Intensive Care and Pain Medicine, Helsinki University and Helsinki University Hospital, Helsinki, Finland; 2 Department of General Practice and Primary Health Care, University of Helsinki and Helsinki University Hospital, Helsinki, Finland; 3 Wellbeing Services County of Vantaa and Kerava, Vantaa and Kerava, Finland; 4 SLEEPWELL Research Programme, Faculty of Medicine, University of Helsinki, Helsinki, Finland

**Keywords:** Fibromyalgia, Diagnostic markers

## Abstract

Fibromyalgia (FM) is associated with sympathetically dominant dysautonomia, but the connection between dysautonomia and FM symptoms is unclear. Dysautonomia can be analysed with heart rate variability (HRV) and it has been proposed that FM patients comprise subgroups with differing profiles of symptom severity. In our study, 51 female FM patients aged 18 to 65 years and 31 age-matched healthy female controls followed a 20-min protocol of alternating relaxation and cognitive stress (mental arithmetic). Heart rates and electrocardiograms were registered. The HRV measures of heart rate (HR), mean interval between heart beats (RR_mean_), root mean squared interval differences of successive beats (RMSSD), and the standard deviation of intervals between normal heart beats (SDNN) were analysed with generalized linear modelling. Features in HRV reactivity which differed between FM patients and controls were used to cluster the FM patients and cluster characteristics were analysed. FM patients had higher baseline HR (72.3 [SD 12.7] vs 64.5 [7.80], p < 0.001) and lower RR_mean_ (0.844 [0.134] vs 0.934 [0.118], p = 0.002), compared with controls. They also reacted to repeated cognitive stress with an attenuated rise in HR (− 4.41 [95% CI − 7.88 to − 0.93], p = 0.013) and attenuated decrease of RR_mean_ (0.06 [95 CI 0.03 to 0.09], p < 0.001), compared with controls. Clustering of FM patients by HRV reactivity resulted in three clusters characterised by (1) normal levels of HRV and HRV reactivity with low levels of depressive mood and anxiety, (2) reduced levels of HRV and impaired HRV reactivity with increased levels of depressive mood and high levels of anxiety, and (3) lowest HRV and most impaired HRV reactivity with the highest scores for depressive mood and anxiety. Our results show that FM patients have lower HRV than healthy controls and their autonomous reactions to cognitive stress are attenuated. Dysautonomia in FM associates with mood disturbance.

**Trial registration** ClinicalTrials.gov (NCT03300635). Registered October 3 2017—Retrospectively registered, https://clinicaltrials.gov/ct2/show/NCT03300635.

## Introduction

Fibromyalgia (FM) is a chronic pain condition which is also characterised by fatigue, sleep disturbance, and subjective cognitive impairment^1,2^. The pathophysiology of FM is largely unclear, though various underlying mechanisms leading to the FM phenotype have been suggested^3,4^.

Autonomic nervous system (ANS) dysfunction is one mechanism that could explain many of the core, as well as other common, symptoms in FM. These include for example dry eyes and mouth, Raynaud’s phenomenon, and orthostatic intolerance^4^. FM patients often have other overlapping functional syndromes, such as irritable bowel syndrome and interstitial cystitis^5–7^. Interestingly, changes in ANS function are also seen in anxiety disorders, which commonly co-occur with FM^8^.

A common method to assess ANS function is heart rate variability (HRV). Heart rate (HR) is constantly regulated by the ANS, with sympathetic nervous system (SNS) (sympathetic) drive increasing, and parasympathetic nervous system (PNS) (vagal) drive decreasing, HR. This ANS balance is in turn affected by the time of the day, breathing, physical activity, emotional state, pain, and many other factors. The interplay between SNS and PNS produces variation in the interval length between heart beats, i.e. HRV. In general, vagal dominance leads to increased, and sympathetic dominance to decreased, HRV^9,10^.

Many studies have found HRV to be reduced in FM, with sympathetic dominance and reduced vagal tone^5,11–14^. FM patients also seem to have a diminished SNS response to stress^13^. Thieme et al. measured psychophysiological responses of FM patients during relaxation and during cognitive and social stress. Based on the measurements, they grouped patients into four clusters^15^. The first (47% of subjects) was characterised by elevated HR and blood pressure (BP), and average skin conductance level (SCL); the second (42%) by decreased BP and BP reactivity, and average SCL; the third (9%) by elevated HR and BP, and high SCL and SCL reactivity; and the fourth (3%) by average HR, BP, and SCL levels and reactivity. As BP and SCL are also controlled by the ANS, this suggests different ANS function patterns in the FM patients.

We hypothesised that by analysing the HRV stress response patterns we could differentiate FM patient clusters and that the clusters would differ in clinical and psychological features. This information could be used as a basis for individualised therapies.

## Methods

We recruited 51 female FM patients aged 18 to 65 years, who fulfilled the American College of Rheumatology 1990 diagnostic criteria for FM^1^, through Helsinki University Hospital (HUH) outpatient clinics, City of Vantaa Health Centre, and the private clinic of one of the authors (RM). Thirty-one healthy, age- and gender-matched controls were also recruited. We recruited the maximum number of patients and matched volunteers available during the funding period of the study and the resulting number was similar to previous studies^11–14^. The exclusion criteria were: diabetes, heart disease, uncontrolled hypertension, peripheral atherosclerotic disease, neurological, neuromuscular or muscle disease, severe psychiatric disorders, continuous use of beta-blockers, beta-agonists, or statins, any musculoskeletal condition that would prevent participation in cycle ergometry (which was to be conducted at a later stage), and poor Finnish language skills that would affect the ability to answer the questionnaires. The patient selection process has been described in detail earlier^16^.

Between November 2015 and June 2018, the subjects visited the HUH Pain Clinic where the diagnosis of FM was confirmed for the patients and excluded for the controls through interview and clinical examination by the same author (TZ). The questionnaires used were completed by the subjects before the measurement protocol described below. We registered our study retrospectively to ClinicalTrials.gov (NCT03300635) on 03/10/2017.

### Background data and questionnaires

We collected data on the subject’s medical background and lifestyle factors. Leisure time physical activity was rated with a four-point Likert scale for frequency (from “None” to “Several times per week”) and for intensity (from “Walking” to “Brisk running”), and subjective physical fitness was rated with a three-point Likert scale (“Worse than average”, “Average”, or “Better than average”). From these, we summed a physical activity score from three to seven. Sleep quality was assessed with a dichotomous question (yes/no) about subjective sleep disturbance and with a four-point Likert scale for waking during sleep (from “Not usually” to “Five or more times per night”). Body mass index (BMI) was calculated from height and weight, and smoking status (smoker/non-smoker) was recorded.

The Fibromyalgia Impact Questionnaire (FIQ) assesses the severity of FM symptoms and their impact on daily functioning^17^. We used the validated Finnish language version of the FIQ, which consists of ten items. These include ability to conduct daily activities; number of days of wellbeing; number of sick leave days; and impact of FM on ability to work during the previous week. The other six items assess the impact of FM on pain intensity, fatigue, feeling refreshed in the mornings, stiffness, anxiety or feeling of tension, and depression or sadness, using visual analogue scales (VAS). The FIQ score ranges from 0 to 100, with higher scores indicating greater FM impact^18^.

The Perceived Stress Scale (PSS) assesses the amount of stress experienced over the previous month, scored from 0 to 40, with higher scores indicating greater stress and less feeling of control^19^. The Pain Catastrophizing Scale (PCS) measures pain-related catastrophizing on a scale from 0 (no catastrophizing) to 52 (most catastrophizing). The State-Trait Anxiety Inventory (STAI) consists of two sub-inventories evaluating immediate feelings of anxiety (state anxiety, STAI-A) and stable, long-term susceptibility to anxiety (trait anxiety, STAI-B). Both STAI-A and STAI-B are scored from 20 to 80, with higher scores indicating more anxiety or anxiety traits respectively.

### Measurement protocol

We modelled our protocol after Thieme et al.^20^, but used only one form of cognitive stress repeatedly, instead of different stressors, to allow evaluation of the effect of repetitive stress. The measurement consisted of five four-minute phases of alternating relaxation and cognitive stress: first relaxation, first stress, second relaxation, second stress, and final relaxation phase.

At the start of the measurement, between each phase, and at the end of the measurement, subjects were asked to rate their subjective stress and pain intensities on a 0 to 10 numeric rating scale (NRS: 0 = no stress or pain; 10 = worst stress or pain imaginable). During the relaxation phases, subjects were asked to relax as best they could while listening to calm classical music or in silence, based on individual preference. During both stress phases, participants were played recordings of 14 series of ten numbers between zero and nine and asked to mentally sum the numbers and state the result. Subjects were told whether their answers were correct or false but were unaware that in four cases they were told that their answer was incorrect, regardless of the actual answer. Throughout the stress phases, background noise of 60 dB white noise was applied.

During the measurements, subjects sat comfortably in a chair in a small room with only one subject and one researcher present. Room temperature was between 20 and 24 °C and humidity between 40 and 60%. As the protocol included speaking, breathing rate was spontaneous and not controlled.

### Heart rate variability measurement and signal processing

Heart rate (HR) as beats per minute was recorded with a heart rate monitor strap (Polar T31, Polar Electro, Finland) placed around the chest at the level of the lowest third of the sternum.

Surface electromyography (sEMG) readings were also recorded bilaterally from the trapezius, biceps, and the erector spinae muscles at L4 level, as published previously^16^. To complement the HR data, we extracted electrocardiogram (ECG) signals from the sEMG recording. We used Matlab R2017b for signal processing. The raw sEMG readings were first detrended to mean zero amplitude. The signals’ power spectra were then inspected visually to identify sharp peaks of alternating current (AC) noise caused by various electronic devices, most commonly at 50 Hz and multiples thereof. Noise peaks were removed using interpolation to flatten the power of the noisy frequency spectrum range of 1 Hz to the mean power of frequency range 0.5 Hz above and below the noisy range.

The best ECG signal was obtained from the right erector spinae (FM n = 24; healthy controls (HC) n = 20) or the left trapezius (FM n = 27; HC n = 11). ECG signals were visually inspected to determine which channel to use for optimum results. A 3-min sample starting at 20 s of each recording was used. From these channels, the QRS complexes were located by filtering the EMG signal with a third-order Butterworth bandpass filter to the 10 to 40 Hz range containing most of the ECG signal. The ECG signal was isolated with principal component analysis (PCA).

HRV analysis was performed using KUBIOS HRV Premium 3.2.0 software. As measures of HRV, we used heart rate (HR), mean interval between heart beats (measured between successive R peaks of the QRS complex in ms [RR]) (RR_mean_), root mean squared interval differences of successive beats (RMSSD), and the standard deviation of intervals between normal heart beats (SDNN). These measures are usable in ultra-short ECG samples, such as the three-minute samples in our case^9^.

For the baseline values, we used the mean HR, RR_mean_, RMSSD, and SDNN for the first 30 s of the recording.

### Statistical methods

For comparison between two groups, the Mann–Whitney U-test was used for continuous variables and the χ^2^-test for categorical variables. Comparisons between three groups or clusters were done with analysis of variance (ANOVA) with *post-hoc* testing using the Tukey test.

We used linear modelling with generalized least squares (GLS) to test how HRV variables were affected by the FM status, the stress-relaxation protocol, and whether the reactions of FM patients and controls differed during the protocol, i.e. group-time interaction. To evaluate reactivity, the base model also needed to include the baseline value of the outcomes. We also tested a second model adjusting for BMI, smoking status, and leisure time physical activity (LTPA). To improve interpretability, LTPA was dichotomised into physically active (physical activity score of seven or more) and inactive. Formulae for HR are shown below:

$$HR \, = \, Group \, \times \, Time \, + \, Baseline,$$

$$HR \, = \, Group \, \times \, Time \, + \, Baseline \, + \, BMI \, + \, Smoking \, + LTPA.$$

Significant group differences in HRV or HRV reactivity were evaluated for suitability as clustering variables for cluster analysis within the FM subgroup post hoc*.* We conducted k-means clustering on z-transformed variables, with the number of variables and clusters restricted to *n* = *10* × *d* × *k* (*d* = number of clustering variables, *k* = number of clusters)^21^.

Statistical analyses were done with R version 4.0.0 (The R Foundation for Statistical Computing 2020). GLS was done with the nlme R package version 3.1-147^22^.

### Ethics approval and consent to participate

The study was conducted in accordance with the Declaration of Helsinki and the study protocol was approved by the Ethics Committee of the Helsinki and Uusimaa Hospital District. All subjects provided written informed consent.

## Results

As previously reported, the FM patients and controls did not differ in age (mean [SD] 45.1 [12.7] vs 46.0 [11.7] years; p = 0.785), but the FM patients had higher BMI (28.2 [5.87] vs 24.7 [3.27] kg/m^2^; p = 0.008) and a tendency to be less physically active (physically active n = 20 [39.2%] vs 16 [51.6%]; p = 0.386) and to smoke (smokers 12 [23.5%] vs 2 [6.5%]; p = 0.091)^16^.

The HRV results by phase are shown in Table 1. The base GLS model (*Outcome* = *Group* × *Time* + *Baseline*) showed that the HRV variables responded to the stress-relaxation protocol with increased HR and decreased RR_mean_ during both stress phases and the intermediary relaxation phase between them, and decreased RMSSD and SDNN during the stress phases. FM patients’ HR response to the second stress phase and RR_mean_ response to the stress phases and the intermediary relaxation phase were lesser than in the controls (Table 2). These differences remained even after adjusting for BMI, smoking, and LTPA (Table 3).

**Table 1:** Heart rate variability measures: heart rate (HR), mean interval between heart beats (RR_mean_), root mean squared interval differences of successive beats (RMSSD), and the standard deviation of intervals between normal heart beats (SDNN).

	HR		RR_mean_		RMSSD		SDNN	
	FM (N = 51)	Control (N = 31)	FM (N = 51)	Control (N = 31)	FM (N = 51)	Control (N = 31)	FM (N = 51)	Control (N = 31)
**Baseline**								
Mean (SD)	72.3 (12.7)	64.5 (7.80)	0.844 (0.134)	0.934 (0.118)	0.0286 (0.0237)	0.0363 (0.0192)	0.0302 (0.0196)	0.0373 (0.0211)
Median [Min, Max]	69.8 [49.1, 115]	63.9 [49.2, 80.6]	0.857 [0.523, 1.21]	0.938 [0.707, 1.24]	0.0254 [0.00260, 0.115]	0.0318 [0.0132, 0.0742]	0.0269 [0.00367, 0.0913]	0.0339 [0.0129, 0.0993]
p	** < 0.001**		**0.002**		0.112		0.136	
**Relaxation phase 1**								
Mean (SD)	73.8 (11.8)	65.9 (8.23)	0.829 (0.125)	0.919 (0.117)	0.0277 (0.0224)	0.0340 (0.0164)	0.0304 (0.0191)	0.0345 (0.0146)
Median [Min, Max]	71.9 [54.6, 113]	65.1 [50.2, 81.9]	0.833 [0.528, 1.13]	0.915 [0.726, 1.19]	0.0244 [0.00246, 0.130]	0.0299 [0.0137, 0.0645]	0.0250 [0.00401, 0.106]	0.0323 [0.0157, 0.0713]
p	** < 0.001**		**0.002**		0.146		0.285	
**Stress phase 1**								
Mean (SD)	82.9 (13.7)	77.5 (12.0)	0.736 (0.130)	0.782 (0.113)	0.0214 (0.0141)	0.0245 (0.0133)	0.0253 (0.0127)	0.0291 (0.0139)
Median [Min, Max]	81.3 [56.0, 116]	75.4 [57.5, 109]	0.730 [0.511, 1.10]	0.775 [0.540, 1.02]	0.0185 [0.00272, 0.0546]	0.0204 [0.00870, 0.0621]	0.0247 [0.00577, 0.0569]	0.0268 [0.0128, 0.0663]
p	0.065		0.099		0.324		0.221	
**Relaxation phase 2**								
Mean (SD)	74.9 (12.0)	68.4 (9.28)	0.822 (0.132)	0.888 (0.123)	0.0255 (0.0168)	0.0310 (0.0165)	0.0291 (0.0155)	0.0342 (0.0150)
Median [Min, Max]	73.6 [56.6, 110]	69.1 [52.3, 88.3]	0.822 [0.540, 1.13]	0.865 [0.670, 1.15]	0.0242 [0.00297, 0.0830]	0.0292 [0.00972, 0.0632]	0.0281 [0.00492, 0.0822]	0.0349 [0.0134, 0.0645]
p	**0.008**		**0.025**		0.153		0.146	
**Stress phase 2**								
Mean (SD)	81.1 (12.9)	77.7 (11.8)	0.750 (0.117)	0.782 (0.115)	0.0212 (0.0152)	0.0246 (0.0137)	0.0254 (0.0146)	0.0277 (0.0118)
Median [Min, Max]	80.4 [58.9, 112]	77.0 [56.8, 112]	0.735 [0.525, 0.991]	0.782 [0.531, 1.06]	0.0180 [0.00274, 0.0675]	0.0232 [0.00695, 0.0651]	0.0223 [0.00541, 0.0675]	0.0265 [0.00916, 0.0623]
p	0.224		0.237		0.313		0.438	
**Relaxation phase 3**								
Mean (SD)	73.7 (11.5)	66.5 (8.00)	0.825 (0.126)	0.913 (0.113)	0.0304 (0.0336)	0.0320 (0.0153)	0.0326 (0.0258)	0.0325 (0.0123)
Median [Min, Max]	73.2 [54.8, 110]	67.7 [50.9, 81.8]	0.815 [0.545, 1.12]	0.881 [0.733, 1.17]	0.0234 [0.00348, 0.210]	0.0296 [0.0105, 0.0682]	0.0268 [0.00582, 0.164]	0.0322 [0.0151, 0.0580]
p	**0.001**		**0.002**		0.779		0.974	

Student’s t-test, p < 0.05.

Significant values are in bold.

**Table 2:** Generalized linear models for heart rate (HR), mean interval between heart beats (RR_mean_), root mean squared interval differences of successive beats (RMSSD), and the standard deviation of intervals between normal heart beats (SDNN), comparing fibromyalgia patients (FM) and healthy controls, unadjusted for lifestyle factors.

Predictors	HR			RR_mean_			RMSSD			SDNN		
	Estimates	CI	p	Estimates	CI	p	Estimates	CI	p	Estimates	CI	p
(Intercept)	6.68	4.55 to 8.81	** < 0.001**	0.07	0.04 to 0.10	** < 0.001**	0.01	0.01 to 0.02	** < 0.001**	0.01	0.01 to 0.02	** < 0.001**
Baseline	0.92	0.89 to 0.95	** < 0.001**	0.91	0.88 to 0.94	** < 0.001**	0.60	0.54 to 0.65	** < 0.001**	0.60	0.55 to 0.65	** < 0.001**
Group [FM]	0.67	− 0.30 to 1.65	0.177	− 0.01	− 0.02 to 0.00	0.170	− 0.00	− 0.01 to 0.00	0.395	0.00	− 0.00 to 0.00	0.916
Time^2^, stress 1	11.56	8.73 to 14.40	** < 0.001**	− 0.14	− 0.16 to − 0.11	** < 0.001**	− 0.01	− 0.01 to − 0.00	** < 0.001**	− 0.01	− 0.01 to − 0.00	**0.034**
Time^3^, relaxation 2	2.47	0.91 to 4.03	**0.002**	− 0.03	− 0.05 to − 0.01	** < 0.001**	− 0.00	− 0.01 to 0.00	0.273	− 0.00	− 0.00 to 0.00	0.910
Time^4^, stress 2	11.74	9.11 to 14.38	** < 0.001**	− 0.14	− 0.16 to − 0.11	** < 0.001**	− 0.01	− 0.01 to − 0.00	**0.001**	− 0.01	− 0.01 to − 0.00	**0.005**
Time^5^, relaxation 3	0.60	− 0.86 to 2.05	0.424	− 0.01	− 0.02 to 0.01	0.459	− 0.00	− 0.01 to 0.01	0.687	− 0.00	− 0.01 to 0.01	0.606
FM: stress 1	− 2.44	− 6.03 to 1.15	0.184	0.04	0.01 to 0.08	**0.012**	0.00	− 0.00 to 0.01	0.306	0.00	− 0.01 to 0.01	0.938
FM: relaxation 2	− 1.38	− 3.36 to 0.59	0.171	0.02	0.00 to 0.05	**0.030**	0.00	− 0.01 to 0.01	0.807	− 0.00	− 0.01 to 0.00	0.706
FM: stress 2	− 4.45	− 7.79 to − 1.11	**0.009**	0.06	0.03 to 0.09	** < 0.001**	0.00	− 0.00 to 0.01	0.386	0.00	− 0.00 to 0.01	0.574
FM: relaxation 3	− 0.69	− 2.54 to 1.17	0.468	0.00	− 0.02 to 0.02	0.830	0.00	− 0.01 to 0.02	0.456	0.00	− 0.01 to 0.01	0.392
Observations	410			410			410			410		

Significant values are in bold.

**Table 3:** Generalized linear models for heart rate (HR), mean interval between heart beats (RR_mean_), root mean squared interval differences of successive beats (RMSSD), and the standard deviation of intervals between normal heart beats (SDNN), comparing fibromyalgia patients (FM) and healthy controls, adjusted for lifestyle factors.

Predictors	HR			BBI			RMSSD			SDNN		
	Estimates	CI	p	Estimates	CI	p	Estimates	CI	p	Estimates	CI	p
(Intercept)	8.31	5.41 to 11.21	** < 0.001**	0.03	− 0.02 to 0.07	0.242	0.02	0.01 to 0.03	** < 0.001**	0.02	0.01 to 0.03	** < 0.001**
Baseline	0.93	0.90 to 0.96	** < 0.001**	0.93	0.90 to 0.96	** < 0.001**	0.60	0.54 to 0.65	** < 0.001**	0.60	0.55 to 0.66	** < 0.001**
Group [FM]	0.85	− 0.20 to 1.89	0.113	− 0.00	− 0.02 to 0.01	0.446	− 0.00	− 0.01 to 0.00	0.309	− 0.00	− 0.00 to 0.00	0.830
Time^2^, stress 1	11.45	8.54 to 14.36	** < 0.001**	− 0.14	− 0.17 to − 0.11	** < 0.001**	− 0.01	− 0.01 to − 0.00	**0.001**	− 0.00	− 0.01 to 0.00	0.059
Time^3^, relaxation 2	2.46	0.85 to 4.07	**0.003**	− 0.03	− 0.05 to − 0.02	** < 0.001**	− 0.00	− 0.01 to 0.00	0.278	− 0.00	− 0.01 to 0.00	0.836
Time^4^, stress 2	11.67	8.92 to 14.41	** < 0.001**	− 0.14	− 0.16 to − 0.11	** < 0.001**	− 0.01	− 0.01 to − 0.00	**0.002**	− 0.01	− 0.01 to − 0.00	**0.010**
Time^5^, relaxation 3	0.68	− 0.85 to 2.20	0.386	− 0.01	− 0.03 to 0.01	0.366	− 0.00	− 0.01 to 0.01	0.701	− 0.00	− 0.01 to 0.01	0.617
BMI (kg/m^2^)	− 0.07	− 0.15 to 0.01	0.091	0.00	− 0.00 to 0.00	0.223	− 0.00	− 0.00 to 0.00	0.169	− 0.00	− 0.00 to 0.00	0.111
Smoker	− 1.45	− 2.45 to − 0.45	**0.005**	0.02	0.01 to 0.03	**0.001**	0.00	− 0.00 to 0.01	0.109	0.00	− 0.00 to 0.00	0.405
Physically active	− 0.79	− 1.65 to 0.06	0.070	0.02	0.01 to 0.02	**0.001**	− 0.00	− 0.00 to 0.00	0.203	− 0.00	− 0.01 to − 0.00	**0.044**
FM: stress 1	− 2.41	− 6.09 to 1.27	0.200	0.05	0.01 to 0.08	**0.006**	0.00	− 0.00 to 0.01	0.446	− 0.00	− 0.01 to 0.01	0.919
FM: relaxation 2	− 1.34	− 3.39 to 0.70	0.198	0.02	0.00 to 0.05	**0.023**	0.00	− 0.01 to 0.01	0.832	− 0.00	− 0.01 to 0.00	0.713
FM: stress 2	− 4.41	− 7.88 to − 0.93	**0.013**	0.06	0.03 to 0.09	** < 0.001**	0.00	− 0.00 to 0.01	0.576	0.00	− 0.01 to 0.01	0.727
FM: relaxation 3	− 0.79	− 2.72 to 1.14	0.424	0.00	− 0.02 to 0.03	0.732	0.01	− 0.01 to 0.02	0.462	0.00	− 0.01 to 0.01	0.404
Observations	385			385			385			385		

Significant values are in bold.

For the k-clustering with our sample size of 51 FM patients, in accordance with *n* = *10* × *d* × *k*, we were limited to two clustering variables and two clusters, or one clustering variable and two or three clusters. As both HR and RR_mean_ reactivity to the second stress phase differed between FM patients and controls, we chose the changes of HR and RR_mean_ from baseline to the second stress phase as the clustering variables (∆HR = HR_stress2_ − HR_baseline_ and ∆RR_mean_ = RR_mean stress2_ − RR_mean baseline_). We tested clustering with ∆HR or ∆RR_mean_ alone, and with ∆HR and ∆RR_mean_. Minimum sum of squares was achieved with ∆RR_mean_ and three clusters, and so we chose this clustering.

We compared the FIQ total score and individual items, PCS, PSS, and STAI scores between the three clusters. FIQ items 3 (days on sick leave) and 4 (impact on ability to work) were excluded due to high percentage of missing data. The HRV, pain, and stress reactivity of the clusters were compared with a sparse GLS model *Variable* = *Cluster* × *Time* + *Baseline.*

Cluster 1 comprised 9 (17.6%) FM patients and was characterised by low baseline HR and stress levels and high baseline RR_mean_, RMSSD, and SDNN with adequate stress responses similar to healthy controls. They had the lowest pain intensity throughout the protocol and low FIQ scores for anxiety and depression.

Cluster 2 comprised 21 (41.2%) FM patients. Their RR and RR_mean_ baseline values and reactivity were intermediate between clusters 1 and 3. They also had high baseline stress and low baseline RMSSD and SDNN with impaired RMSSD reactivity and high pain intensity similar to cluster 3. Their FIQ scores for anxiety were high with a tendency for higher depression scores.

Finally, Cluster 3 comprised 21 (41.2%) FM patients and was characterised by high baseline HR and stress and low baseline RR_mean_, RMSSD, and SSDN with low HRV reactivity, and high pain intensity throughout the protocol. They had high FIQ scores for anxiety and the highest depression scores of the clusters.

**Figure 1:**
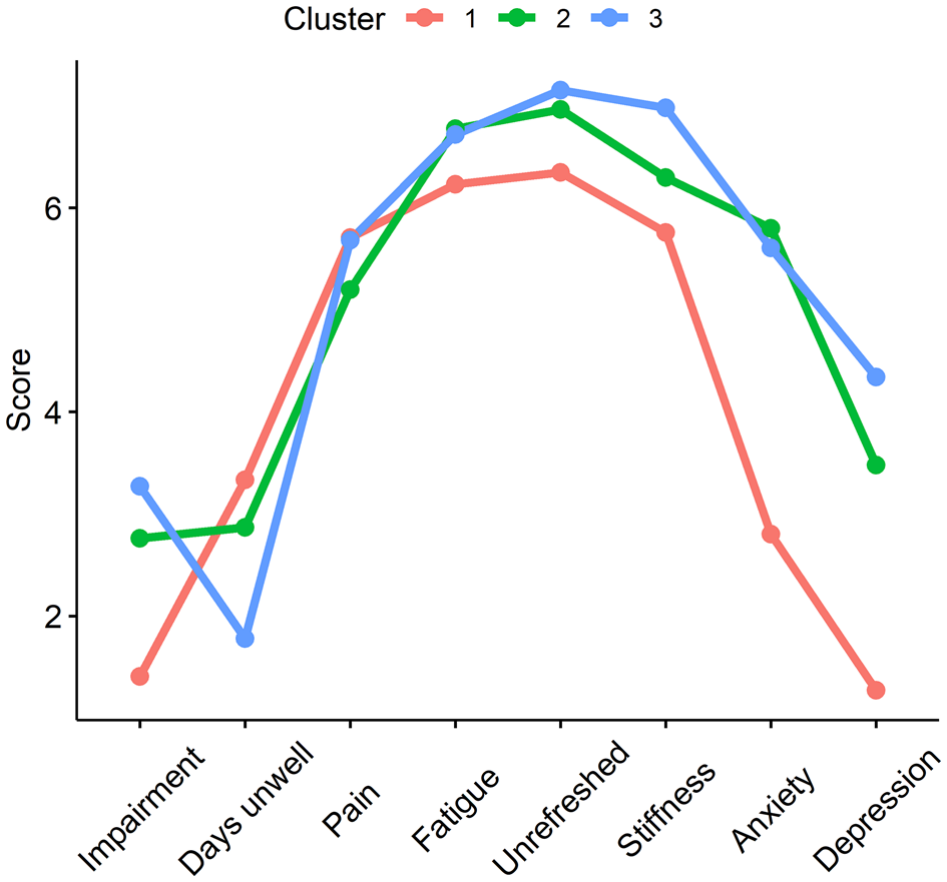
Mean scores of individual items in the Fibromyalgia Impact Questionnaire in the three clusters of the fibromyalgia patients. Item 1 (Impairment): Were you able to (subitems A–J: do shopping, prepare meals, etc.)?; Item 2 (Days unwell): Of the 7 days in the past week, how many days did you feel good? (higher score = more days unwell); Item 5 (Pain): How bad has your pain been? (visual analogue scale [VAS]); Item 6 (Fatigue): How tired have you been? (VAS); Item 7 (Unrefreshed): How have you felt when you get up in the morning? (VAS); Item 8 (Stiffness): How bad has your stiffness been? (VAS); Item 9 (Anxiety): How nervous, or anxious have you felt? (VAS); Item 10 (Depression): How depressed or blue have you felt? (VAS). Significant differences in Anxiety between clusters 2 and 3 compared with cluster 1, and Depression between cluster 3 compared with cluster 1 at p < 0.05 (Tukey’s HSD).

**Figure 2:**
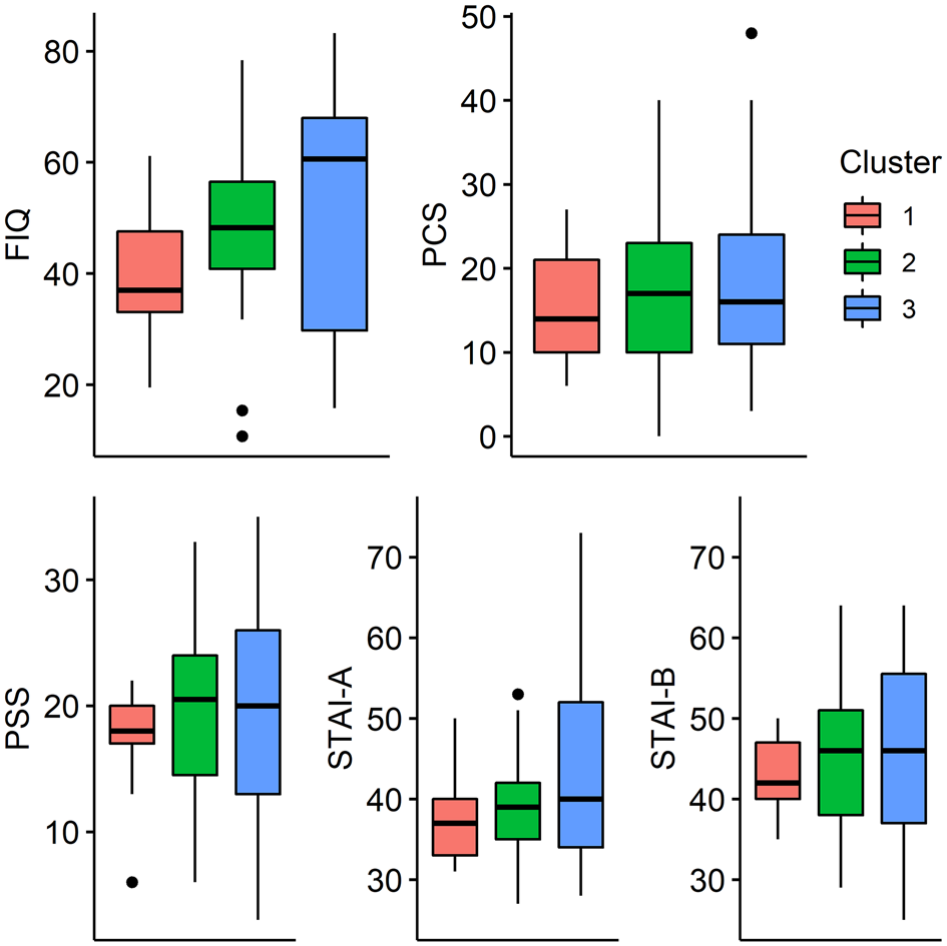
Box plots of the fibromyalgia impact questionnaire (FIQ), pain catastrophizing scale (PCS), perceived stress scale (PSS), and state-trait anxiety inventory A (state anxiety, STAI-A) and B (trait anxiety, STAI-B), scores in the three clusters of fibromyalgia patients.

**Figure 3:**
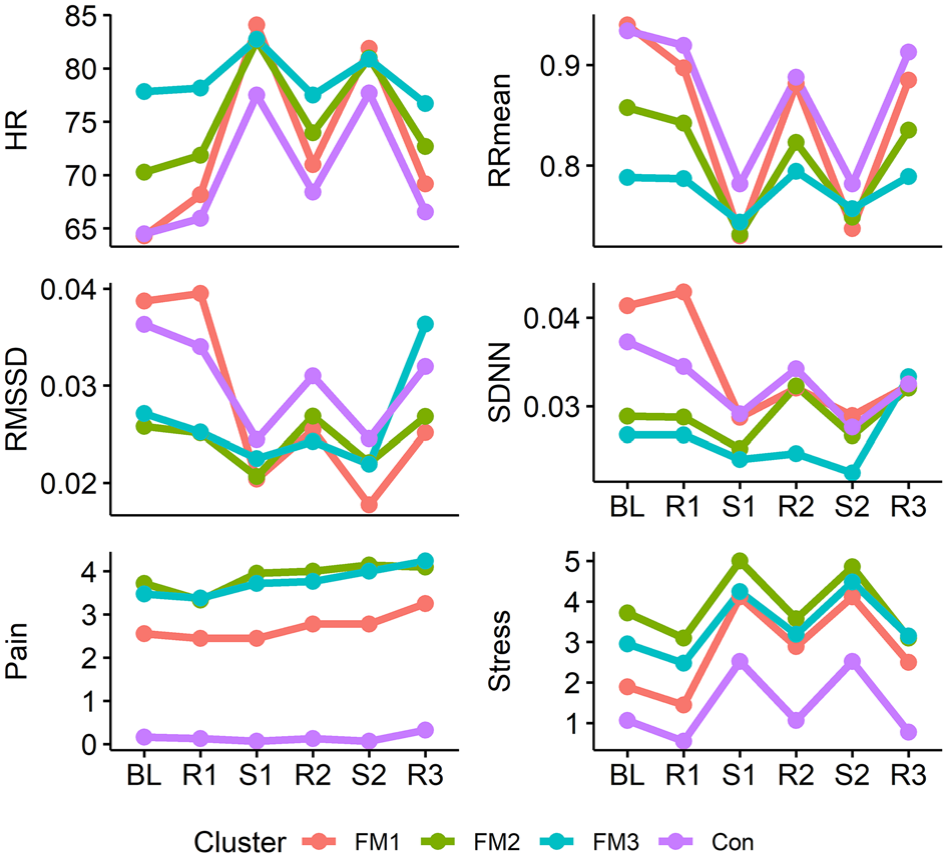
Mean heart rate (HR, beats per minute), RMSSD (ms), subjective pain (numeric rating scale [NRS] from 0 to 10), and subjective stress (NRS from 0 to 10) levels during the measurement in the three fibromyalgia patient clusters (FM1, 2, 3) and healthy controls (Con). *BL* baseline, *R1* first relaxation phase, *S1* first cognitive stress phase, *R2* second relaxation phase, *S2* second cognitive stress phase, *R3* third and final relaxation phase.

Pain reactivity to cognitive stress did not differ between clusters. The clusters did not differ by age, BMI, smoking status, or LTPA, though cluster 1 showed a tendency for lower BMI (Figs. 1, 2, 3, Table 4).

**Table 4:** Generalized linear models for heart rate (HR), mean interval between heart beats (RR_mean_), root mean squared interval differences of successive beats (RMSSD), and the standard deviation of intervals between normal heart beats (SDNN), and subjective pain and stress, comparing the three fibromyalgia patient clusters.

Predictors	HR			RR_mean_			RMSSD			SDNN			Pain			Stress		
	Estimates	CI	p	Estimates	CI	p	Estimates	CI	p	Estimates	CI	p	Estimates	CI	p	Estimates	CI	p
(Intercept)	6.82	4.36–9.28	** < 0.001**	− 0.00	− 0.03 to 0.03	0.955	0.02	0.01 to 0.03	** < 0.001**	0.01	0.01 to 0.02	** < 0.001**	0.26	− 0.45 to 0.97	0.474	− 0.32	− 0.85 to 0.20	0.227
Baseline	0.95	0.92 to 0.99	** < 0.001**	0.96	0.92 to 0.99	** < 0.001**	0.56	0.49 to 0.63	** < 0.001**	0.68	0.61 to 0.75	** < 0.001**	0.86	0.78 to 0.93	** < 0.001**	0.94	0.87 to 1.00	** < 0.001**
Cluster^2^	− 2.00	− 3.54 to − 0.45	**0.012**	0.02	0.01 to 0.04	**0.005**	− 0.01	− 0.02 to 0.00	0.092	− 0.01	− 0.01 to 0.00	0.063	− 0.10	− 0.92 to 0.72	0.807	− 0.06	− 0.68 to 0.56	0.855
Cluster^3^	− 2.92	− 4.51 to − 1.33	** < 0.001**	0.03	0.02 to 0.05	** < 0.001**	− 0.01	− 0.02 to 0.00	0.065	− 0.01	− 0.01 to − 0.00	**0.040**	0.15	− 0.67 to 0.97	0.721	0.04	− 0.58 to 0.65	0.908
Time^2^, stress 1	15.91	11.70 to 20.13	** < 0.001**	− 0.17	− 0.20 to − 0.13	** < 0.001**	− 0.02	− 0.03 to − 0.01	** < 0.001**	− 0.01	− 0.02 to − 0.01	**0.002**	− 0.00	− 1.19 to 1.19	1.000	2.67	1.64 to 3.70	** < 0.001**
Time^3^, relaxation 2	2.83	0.38 to 5.28	**0.025**	− 0.02	− 0.04 to 0.01	0.229	− 0.01	− 0.03 to − 0.00	**0.017**	− 0.01	− 0.02 to − 0.00	**0.010**	0.33	− 0.82 to 1.48	0.571	1.44	0.43 to 2.46	**0.006**
Time^4^, stress 2	13.72	10.32 to 17.12	** < 0.001**	− 0.16	− 0.18 to − 0.14	** < 0.001**	− 0.02	− 0.03 to − 0.01	** < 0.001**	− 0.01	− 0.02 to − 0.01	**0.002**	0.33	− 0.89 to 1.56	0.595	2.67	1.30 to 4.03	** < 0.001**
Time^5^, relaxation 3	1.01	− 1.42 to 3.43	0.417	− 0.01	− 0.04 to 0.02	0.448	− 0.01	− 0.04 to 0.01	0.218	− 0.01	− 0.03 to 0.01	0.217	0.53	− 0.79 to 1.85	0.430	0.83	− 0.37 to 2.04	0.177
Cluster^2^ : stress 1	− 5.14	− 10.18 to − 0.10	**0.047**	0.06	0.01 to 0.10	**0.012**	0.01	0.00 to 0.03	**0.012**	0.01	0.00 to 0.02	**0.049**	0.62	− 0.81 to 2.04	0.395	− 0.76	− 1.99 to 0.47	0.226
Cluster^3^ : stress 1	− 11.34	− 16.38 to − 6.31	** < 0.001**	0.12	0.08 to 0.17	** < 0.001**	0.02	0.01 to 0.03	**0.005**	0.01	0.00 to 0.02	**0.034**	0.33	− 1.09 to 1.76	0.647	− 0.90	− 2.13 to 0.33	0.151
Cluster^2^ : relaxation 2	− 0.74	− 3.67 to 2.19	0.620	− 0.00	− 0.03 to 0.03	0.822	0.02	0.00 to 0.03	**0.025**	0.01	0.00 to 0.02	**0.004**	0.33	− 1.04 to 1.71	0.635	− 0.97	− 2.18 to 0.24	0.119
Cluster^3^ : relaxation 2	− 3.50	− 6.43 to − 0.57	**0.020**	0.02	− 0.01 to 0.05	0.142	0.01	− 0.00 to 0.03	0.061	0.01	− 0.00 to 0.02	0.078	0.05	− 1.33 to 1.42	0.946	− 0.73	− 1.94 to 0.48	0.239
Cluster^2^ : stress 2	− 4.61	− 8.67 to − 0.54	**0.027**	0.07	0.04 to 0.09	** < 0.001**	0.02	0.01 to 0.03	**0.006**	0.01	0.00 to 0.02	**0.023**	0.48	− 0.99 to 1.94	0.525	− 0.90	− 2.54 to 0.73	0.279
Cluster^3^ : stress 2	− 11.00	− 15.07 to − 6.94	** < 0.001**	0.13	0.10 to 0.16	** < 0.001**	0.02	0.01 to 0.03	**0.006**	0.01	− 0.00 to 0.02	0.063	0.29	− 1.18 to 1.75	0.703	− 0.67	− 2.30 to 0.97	0.425
Cluster^2^ : relaxation 3	− 0.20	− 3.11 to 2.70	0.890	0.01	− 0.03 to 0.04	0.786	0.02	− 0.01 to 0.04	0.250	0.01	− 0.01 to 0.03	0.176	0.23	− 1.33 to 1.79	0.773	− 0.83	− 2.26 to 0.59	0.252
Cluster^3^ : relaxation 3	− 2.46	− 5.36 to 0.44	0.098	0.01	− 0.02 to 0.05	0.459	0.03	− 0.00 to 0.05	0.068	0.02	− 0.00 to 0.04	0.094	0.32	− 1.23 to 1.88	0.683	− 0.17	− 1.59 to 1.26	0.817
Observations	255			255			255			255			254			254		

Significant values are in bold.

## Discussion

Overall, we found FM patients to have higher heart rates and lower HRV, compared with healthy controls. The HRV stress responses were also attenuated in FM patients, compared with controls, and this attenuation was more pronounced with repetition of cognitive stress.

It is unclear how precisely PNS and SNS activity can be deduced from HRV measures. The low frequency (LF) to high frequency (HF) ratio (LF/HF) has been extensively used as a measure of SNS/PNS balance, but this has now been questioned^23^. RMSSD is currently considered the best measure of PNS activity^24^. RMSSD values we measured were valid with baseline values adequately within normative values as reported by Tegegni et al.^25^ (96.1% and 100% within the 98th centile and 90.2% and 74.2% within the 75th centile for FM patients and controls respectively). However, RMSSD did not differ between patients and controls, suggesting differences in SNS activity primarily responsible for differences in their HRV. However, the patients with more pronounced dysautonomia (clusters 2 and 3) also had lower baseline RMSSD. Therefore, we hypothesise that both PNS and SNS abnormalities are involved.

Our findings are in line with published studies, with most showing sympathetic dominance in FM. The 2014 systematic review by Martínez-Martínez et al. included 70 studies of ANS function in FM, of which 47 reported sympathetic dominance, with only 4 reporting parasympathetic dominance, whereas 9 found no difference between FM and controls and 10 reported dysautonomia while not specifying sympathetic or parasympathetic dominance^5^. It should nevertheless be noted that publication bias may be reflected in the small number of negative findings.

After analysing the main outcomes, we clustered the FM patients in an exploratory manner, based on the most prominent difference between FM patients and controls in our results, namely the RR_mean_ stress response. This resulted in three distinct clusters of FM patients.

The main features of the first cluster were baseline HRV parameters and autonomic stress responses similar to those of the healthy controls, i.e. no ANS dysfunction. They had the lowest symptom scores for anxiety and depression and tended to have lower pain intensity than the other clusters.

The second cluster had lower baseline HRV which was intermediate between healthy controls or Cluster 1 and Cluster 3, with HRV reactivity also retained under the stress-relaxation protocol. This less severe form of ANS dysfunction associated with high symptom scores for anxiety and somewhat elevated scores for depression.

The third cluster had the lowest baseline HRV and attenuated stress responses. This could be described as severe ANS dysfunction with loss of stress reactivity. The FM patients in this cluster had the highest scores for both anxiety and depressive mood in the FM cohort.

Reyes del Paso et al. also measured HRV reactivity during rest and an arithmetic task and found blunted autonomic responses to stress in conjunction with increased pain severity. They reported that the HRV measured with RR was lower among FM patients than controls, with depression correlating to lower HRV, which is similar to Cluster 3 in our study. The patients in Cluster 3 had higher scores for depressive mood but, unlike the findings of Reyes del Paso et al., anxiety did not correlate with greater HRV reactivity in Cluster 3 or Cluster 2 patients. Reyes del Paso et al. also measured blood pressure and linked the increased pain sensitivity to decreased baroreflex sensitivity. Unfortunately, we could not compare blood pressure reactions, as we did not measure blood pressure^11^.

Compared with our findings, Thieme et al. used a greater number of clustering variables in addition to HR and HR reactivity and found four distinct clusters. Their Cluster 1 shares some similarities with our Cluster 1, showing the greatest HR and pain reactivity to mental arithmetic and the lowest levels of stress. Unlike our Cluster 3, their Cluster 1 was larger (47%) and had intermediate levels of anxiety. Thieme et al.’s Clusters 3 and 4 (9% and 3% respectively) shared features with our Cluster 3, with their Cluster 3 having elevated depression scores and their Cluster 4 elevated anxiety scores, and both showing decreased HR reactivity to mental arithmetic. However, it is unclear to what extent the clusters identified by Thieme et al. overlap with the clusters we identified. Their patient population was also female and of similar age, but other demographic factors along with the difference in clustering variables may have contribute to the difference in cluster sizes^15^.

Psychological symptoms are often involved in FM, as this and previous studies show, and pain treatment should include their management, with e.g., cognitive behavioural therapy intervention^26^. However, the findings about ANS function when comparing FM patients with healthy controls, and within FM patients in respect to anxiety and depression symptoms, raise the question of whether ANS dysfunction acts as a shared factor in both FM and psychological symptoms. This may provide a novel perspective for treatment. Identifying shared processes across pain conditions and comorbid problems is one primary objective in seeking improved pain treatment outcomes^27^. Decreased HRV is seen in both anxiety and depression and also in conjunction with worry at levels below the diagnosis of clinical anxiety disorder^8,28^. Interventions which appear to benefit ANS function, such as HRV biofeedback, relaxation training, and mindfulness-based techniques^28–30^, might then be especially useful in the treatment of FM.

Monoamines are involved in both ANS function and pain modulation. The metabolism of catecholamines in the prefrontal cortex depends on the activity of catechol-O-methyltransferase (COMT)^31^. It has been suggested that COMT gene variants associated with decreased pain modulation would be more frequent in FM patients^32^.

While we found that attenuated autonomic responses to cognitive stress correlate with anxiety and depressive mood in fibromyalgia patients, there are several limitations to be considered. HRV and autonomic stress responses are known to be affected by gender and sex hormone status^33^. As our study subjects were female, which eliminated confounding by gender, these results should not be generalised to male patients without further study. Also, we did not assess the hormonal status of our study subjects (menstrual phase, use of hormonal contraception, menopause, etc.). Stress responses could also appear attenuated, if the induction of the experimental subjective stress was unsuccessful. The stress intensities reported suggest that this was not a problem with our results, but this is an important consideration in the design of future studies. The exploratory nature of our statistical method may increase the risk of chance findings. However, our findings are logical and in line with previous studies. Further studies based on our findings are warranted.

## Conclusions

Our results support previous findings of sympathetic dominance and attenuated ANS stress response in FM, compared with healthy persons. Due to the limited sample size and exploratory nature of our study, our clustering must be considered tentative. However, greater FM impact characterised by feelings of anxiety and depressive mood could be connected to ANS dysfunction in a portion of FM patients, with worse dysfunction impairing ANS stress responsiveness. Treatment of symptoms of anxiety or depression in FM patients having such symptoms could improve their resilience to stressful factors and reduce FM flare-ups. The possibility of treating mood disturbance in FM by interventions strengthening the autonomous nervous system should also be considered.

## Data Availability

The datasets generated and analysed during the current study are not publicly available as consent for publication was not asked from the study subjects. The data are available from the corresponding author on reasonable request if also approved by our ethics committee.

## Abbreviations

AC: Alternating current
ANOVA: Analysis of variance
ANS: Autonomic nervous system
BMI: Body mass index
BP: Blood pressure
CI: Confidence interval
COMT: Catechol-O-methyltransferase
ECG: Electrocardiogram
FIQ: Fibromyalgia impact questionnaire
FM: Fibromyalgia
GLS: Generalized least squares
HC: Healthy control
HR: Heart rate
HRV: Heart rate variability
HUH: Helsinki University Hospital
LTPA: Leisure time physical activity
NRS: Numerical rating scale
PCA: Principal component analysis
PCS: Pain catastrophizing scale
PNS: Parasympathetic nervous system
PSS: Perceived stress scale
RMSSD: Root mean squared interval differences of successive beats
RR: Interval of successive heart beats
SCL: Skin conductance level
SD: Standard deviation
sEMG: Surface electromyography
SNS: Sympathetic nervous system
STAI: State-trait anxiety inventory
STAI-A: State-trait anxiety inventory, state anxiety subinventory
STAI-B: State-trait anxiety inventory, trait anxiety subinventory
VAS: Visual analogue scale
